# Exploring the Immune System's Role in Endometriosis: Insights Into Pathogenesis, Pain, and Treatment

**DOI:** 10.7759/cureus.87091

**Published:** 2025-07-01

**Authors:** Rania S Ahmed, Mohamed Sherif, Majd A Alghamdi, Salah N El-Tallawy, Omar K Alzaydan, Joseph V Pergolizzi, Giustino Varrassi, Zaina Zaghra, Ziad S Abdelsalam, Mahmoud T Kamal, Flaminia Coluzzi

**Affiliations:** 1 College of Medicine, Alfaisal University College of Medicine, Riyadh, SAU; 2 Anesthesia and Pain Management, Faculty of Medicine, Minia University, Minia, EGY; 3 College of Medicine, King Saud University, King Abdulaziz University Hospital, Riyadh, SAU; 4 Anesthesia and Pain Management, National Cancer Institute, Cairo University, Cairo, EGY; 5 Obstetrics and Gynecology, King Khalid University Hospital, Riyadh, SAU; 6 Pain Medicine, NEMA Research Inc, Naples, USA; 7 Pain Medicine, Fondazione Paolo Procacci, Rome, ITA; 8 College of Medicine, Bahcesehir University, Istanbul, TUR; 9 Otolaryngology, Minia University, Minia, EGY; 10 Obstetrics and Gynecology, Minia University Hospital, Minia, EGY; 11 Medical and Surgical Sciences, Sapienza University of Rome, Rome, ITA

**Keywords:** adaptive immune system, biomarker, endometriosis, endometriosis pain, innate immune system, neuroinflammation

## Abstract

Endometriosis is a common gynecological condition that usually affects women during their reproductive years. The main goal of this narrative review is to understand the role of the innate and adaptive immune system in the pathogenesis of endometriosis and to explore ways to diagnose and treat endometriosis effectively. Methodology: A comprehensive literature search was conducted across PubMed, EMBASE, Cochrane, and Scopus. Full-text articles in English published between January 2019 and January 2025 were included in this review. Two independent reviewers screened titles and abstracts using preset inclusion and exclusion criteria, as well as relevant keywords related to endometriosis, immune responses, and chronic pain. Of 6,728 initially identified studies, 53 met the inclusion criteria after applying exclusion parameters. Recent papers shed light on the role of the immune system in the pathogenesis of endometriosis. The dysregulation of immune system cells, such as macrophages, natural killer cells, neutrophils, dendritic cells, T cells, and B cells, contributes to the pathogenesis of endometriosis by enhancing the development and progression of endometriosis lesions, thereby creating an inflammatory environment. The presence of the inflammatory environment not only supports the survival and growth of the ectopic endometrial lesion but also plays a vital role in the pain associated with it. Endometriosis is a disease characterized by the presence of ectopic endometrial-like tissue outside the uterine cavity. It is believed that this ectopic endometrial tissue is a result of an interplay between hormonal, immune, and environmental factors. To this day, the gold standard for diagnosing endometriosis is through laparoscopic surgery, and there is no long-lasting effective medical treatment.

## Introduction and background

Endometriosis is a gynecological disease characterized by the ectopic seeding of endometrial tissue outside the uterine cavity [1,2]. It commonly presents with symptoms such as chronic pelvic pain, dysmenorrhea (painful menstruation), dyspareunia (pain during intercourse), and infertility [3-5]. The ectopic tissue is most often found in the ovaries, fallopian tubes, peritoneum, and bladder but can also appear in extra-pelvic sites [6,7]. The condition affects around 10% of women of reproductive age [8]. Endometrial tissue situated outside the uterine cavity is theorized to undergo inflammatory changes that can further complicate the condition [9], with T helper 17 cells (Th17) cell-mediated induction pathways [10], IL-17-mediated pain mechanisms [11], and dendritic cell modulation induced by IL-17 [12]. Prolonged inflammation is thought to cause adhesions in the pelvic cavity, disrupting the anatomy of the reproductive apparatus and increasing the likelihood of infertility [13,14]. 

The pathophysiology of endometriosis involves a complex interplay between hormonal, genetic, inflammatory, and immune factors [14,15]. Recent research revealed that both arms of the immune system, the innate and the adaptive, are dysregulated and theorized to be active components in the pathophysiology of endometriosis [16,17]. Under normal physiological conditions, immune cells efficiently clear retrograde menstrual debris from the peritoneal cavity. In endometriosis, however, this immune clearance appears to fail. An interplay between natural killer (NK) cells, neutrophils, macrophages, and the cytokines they secrete when stimulated is thought to be responsible for some of the presenting symptoms of endometriosis, such as pain [15,16]. Macrophages exhibit impaired phagocytic capacity [8], while simultaneously releasing pro-inflammatory cytokines and angiogenic factors that support lesion survival and growth [9]. NK cells, which normally target aberrant or infected cells, exhibit diminished cytotoxicity due to the upregulation of inhibitory receptors, thereby allowing ectopic tissue to evade immune surveillance [16].

Additionally, T lymphocytes are skewed toward a tolerogenic phenotype, with an imbalance favoring pro-inflammatory Th17 cells over regulatory T cells (Tregs), which contributes to chronic inflammation and the immune tolerance of the lesions. These immune disturbances promote lesion progression. The unique markers and receptors specific to certain immune cells that are active in endometriosis can act as guides to assess the severity and progression of endometriosis. Pro-inflammatory cytokines and neurotrophic factors released by immune cells sensitize nerve endings and are recognized as central to the pain experienced in endometriosis. Traditionally, endometriosis has been approached through hormonal therapies aimed at suppressing ovarian function or via surgical excision of lesions. However, these strategies are often symptomatic rather than curative [16,17].

This narrative review aims to explore the immunological dysfunction underlying endometriosis, highlighting how specific components of the immune system contribute to the pathogenesis and symptomatology of endometriosis. Additionally, it explains how these insights may facilitate earlier diagnosis and more effective management strategies.

## Review

Methods

**Figure 1:**
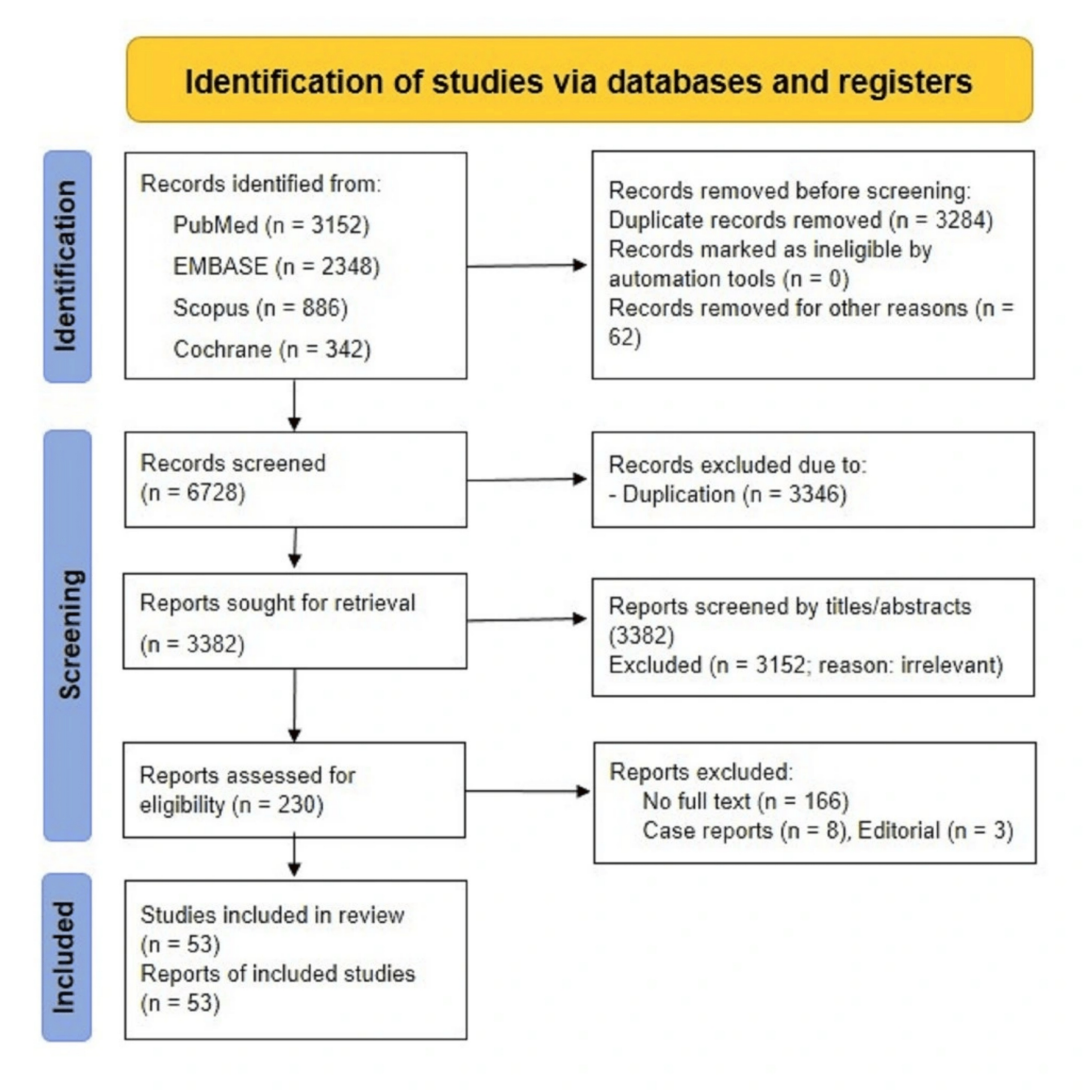
PRISMA chart of the included studies

An extensive literature search was conducted using sources like PubMed, EMBASE, Scopus, and Cochrane databases. The search strategy included articles that were published during the last six years, between January 2019 and May 2025. The search strategy included original research articles, and peer-reviewed studies such as observational, cross-sectional, cohort, longitudinal studies, systematic reviews, and practice guidelines. Articles that met the inclusion criteria, such as articles published in English, were available in full text, relevant to the condition, and presented information on endometriosis (e.g., mechanisms, diagnostic, or therapeutic aspects), the immune system, and chronic pain due to endometriosis, were included in this review. The exclusion criteria included any paper outside the specified time range, non-English publications, articles not available as free full text, and those that were case reports, case series, or editorial letters. The search was performed by two independent reviewers who filtered the papers by reading the title and abstract to determine that they met the inclusion criteria. The search was conducted using keywords such as "endometriosis," "innate immune system," "adaptive immune system," "biomarker," "neuroinflammation," "endometriosis pain," and "treatment." A total of 6,728 publications were identified through a data search conducted over the past six years. Two independent reviewers screened the studies by title and abstract. Any disagreement in selection was resolved after discussion, ensuring a consistent and unbiased approach to inclusion. After removing 3,346 duplicate or non-English publications, 3,382 articles remained for title and abstract screening. Of these, 3,152 were excluded based on title and abstract review. The remaining 230 full-text publications were assessed for eligibility. Following this, 177 articles were excluded due to the unavailability of full text, case reports, or editorial letters. Ultimately, 53 publications were included in the final article (Figure 1). The SANRA (Scale for the Assessment of Narrative Review Articles) format for reporting narrative reviews is used for evaluating the quality of the manuscript [18]. As this is a narrative review, no statistical analysis or quantitative synthesis was conducted. No ethical considerations were required since this manuscript is based on previous research, and there was no involvement of any animals or human participants.

Overview of endometriosis

Endometriosis is a common benign gynecological condition that affects women of reproductive age [8]. Endometriosis occurs when endometrial-like tissue, containing the gland and stroma, is found outside the uterus [8,9]. Endometriosis affects roughly 10% (190 million) of reproductive-age women and girls globally [9]. The presence of these endometriosis lesions outside the uterine cavity, which react to the hormonal cycle like the tissue in the uterus, causes increased inflammation and release of pain mediators, leading to dysmenorrhea, dyspareunia, and chronic pain [1-5]. After an extensive inflammatory process, fibrosis occurs, which could lead to pelvic adhesions that distort the normal anatomy of the reproductive organs and subsequently lead to infertility [13-15]. This endometrial-like tissue is most commonly found in the pelvic cavity, including the ovary, uterosacral ligaments, pouch of Douglas, bladder, broad ligament, fallopian tube, sigmoid colon, or rectum [6,7]. Moreover, they can be found, although to a lesser extent, in extra-pelvic sites, such as the anterior abdominal wall, sites of surgical incisions, or even in the thoracic cavity [19,20]. Endometriosis is a complex disease in which the interplay between hormonal, immunological, and environmental factors plays a role in its development [10-12]. There have been multiple theories, such as retrograde menstruation (the currently widely accepted theory used in the paper), celomic metaplasia, and the dissemination of endometrial-like tissue through the blood or lymphatic system. The proposed theories attempted to explain the etiology of endometriosis but have not managed to sufficiently explain the disease's etiology [9,21]. The current gold standard diagnostic method of endometriosis is through laparoscopic surgery and a biopsy of the tissue [22-24]. Although not cost-effective, many physicians diagnose patients with endometriosis presumptively through the signs and symptoms of the patient, as well as ultrasound findings, which depend on the physician's skills [22,23]. There are no lab tests, so far, that can confirm the diagnosis of endometriosis through a non-invasive method [9,22]. Since the pathogenesis of endometriosis is quite complex and not clearly understood, there is no effective treatment that can cure endometriosis. All the treatment methods available, including non-steroidal anti-inflammatory drugs (NSAIDs), combined oral contraceptives, hormonal intrauterine devices, vaginal rings, implants, injections, patches, and gonadotropin-releasing hormone agonists (GnRH agonists), are symptomatic rather than curative. In addition, surgical removal of the ectopic tissue is merely symptomatic as it can recur [8,9]. Thus, understanding the immune system and its role in the pathogenesis of endometriosis will help in developing ways to more effectively diagnose and treat endometriosis.

Immune dysregulation in endometriosis

In healthy women, the immune system efficiently eliminates menstrual debris, maintaining a state of homeostasis. However, in endometriosis, this crucial immune function fails [17]. This was further expanded in a single-cell study by Hou et al., which delineated dysfunctional immune subsets, including exhausted NK cells and regulatory-like macrophages, underscoring microenvironmental immunosuppression in endometriosis [25]. This was further expanded in a single-cell study by Hou et al. (2025), which delineated dysfunctional immune subsets, including exhausted NK cells and regulatory-like macrophages, underscoring microenvironmental immunosuppression in endometriosis [26]. Complementing this, Boldu-Fernández et al. (2025) used single-nucleus sequencing to reveal spatially distinct T cell populations in eutopic versus ectopic lesions, with enrichment of IL-17+ and PD-1+ phenotypes in deep infiltrating disease [27]. The immune system fails to effectively recognize and eliminate the ectopic endometrial tissue, allowing it to grow and thrive further [12,16,28]. This failure of immune clearance is caused by the dysregulation of both the innate and adaptive immune cells [17]. Macrophages, key players in the innate response, normally phagocytose the menstrual debris and maintain homeostasis. However, their phagocytic ability as well as the production of pro-inflammatory cytokines and angiogenic factors, which enhance the survival, vascularization, and fibrogenesis of the ectopic tissue, allowing it to implant faster, are decreased in endometriosis [17]. Furthermore, they also interact with the nerve endings to promote the pathophysiology of endometriosis-related pain [29,30]. There is also a decrease in the number and cytotoxic activity of natural killer (NK) cells, another key component of the innate immune system, in the peritoneal fluid of individuals with endometriosis, due to the upregulation of inhibitory receptors. This immune suppression allows ectopic endometrial tissue to evade immune surveillance, facilitating its implantation and subsequent growth [17]. Neutrophils, part of the innate immune system, also play a significant role in the early stages of endometriosis by promoting angiogenesis through vascular endothelial growth factor (VEGF), inflammation, and tissue damage through NETs [17]. The dendritic cells (DCs), also a key component of the innate immune response, aid in angiogenesis and inflammation, but the exact mechanism is still unclear [17]. Furthermore, the adaptive immune system also plays a vital role in the pathogenesis of endometriosis. It has been observed that there is an imbalance between the subsets of T cells in the peritoneal fluid of patients with endometriosis [17]. There is an observed significant downregulation of Tregs, but an upregulation of T helper 17 cells, which contributes to the state of immune tolerance, allowing the endometriosis lesion to persist and grow [17,31-33]. Emerging clinical investigations have targeted the IL-17 pathway to alleviate chronic pelvic pain, building on preclinical evidence showing IL-1β-mediated neurotrophin regulation in endometriotic tissues (Table 1) [18].

**Table 1:** Immune cell roles in endometriosis IL-6 = interleukin-6, TNF-α = tumor necrosis factor-alpha, VEGF = vascular endothelial growth factor, TGF-β1 = transforming growth factor-beta 1, NK cells = natural killer cells, KIR = killer-cell immunoglobulin-like receptor, PD-1 = programmed cell death protein 1, IFN-γ = interferon-gamma, iDC = immature dendritic cells, mDC = mature dendritic cells, Treg = regulatory T cells, Th17 = T helper 17 cells

Immune Cell	Role Description
Macrophages	↑ IL-6, TNF-α ↑ VEGF, TGF-β1 ↓ Phagocytosis (Pro-inflammatory)
NK Cells	↓ Cytotoxicity ↑ KIR/PD-1 ↓ IFN-γ (Suppressed Immunity)
Neutrophils	↑ IL-8, NETs ↑ VEGF (Inflammatory and Angiogenic)
Dendritic Cells	↑ iDC / ↓ mDC ↑ IL-10 ↓ Antigen Presentation (Tolerogenic)
T Cells	↑ Treg, Th17 ↑ IL-10, IL-17 (Mixed Role)
B Cells	↑ Autoantibodies ↑ IL-6, IFN-γ (Autoimmunity and Inflammation)

**Figure 2:**
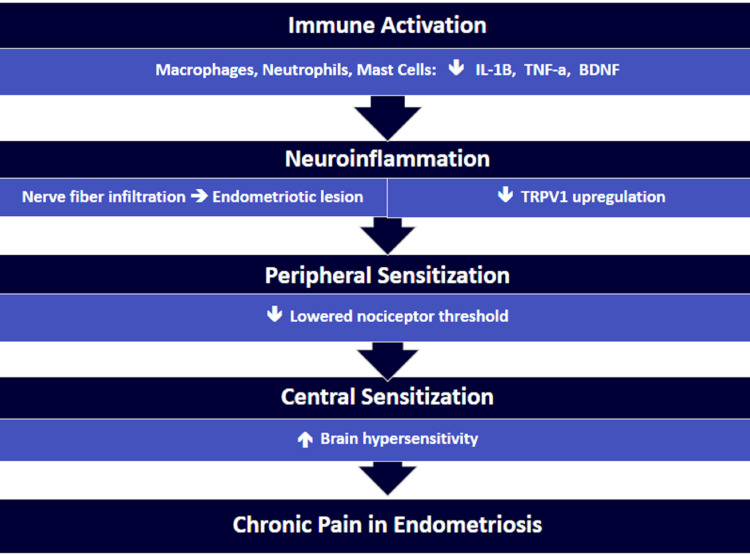
Illustration of the pain mechanism in endometriosis IL-1β = interleukin-1 beta, TNF-α = tumor necrosis factor-alpha, BDNF = brain-derived neurotrophic factor, TRPV1 = transient receptor potential vanilloid 1 Image Credit: Authors

As a result, this chronic, dysregulated inflammatory state sensitizes the nervous system and promotes the growth of the nerve fibers into the endometriosis lesion, which causes amplified activation of the nociceptors, leading to peripheral and central sensitization, and contributes to the pain associated with endometriosis [16,17,34]. More details about how the immune system plays a role in the pathogenesis of endometriosis and how the knowledge regarding the immune system can be applied clinically are discussed in the following section (Figure 2).

Immune markers

Many of the immune markers related to endometriosis have been mentioned in the literature. In the following sections, we will briefly discuss the normal function of these immune markers and how they contribute to the pathogenesis of endometriosis and its related pain. Additionally, we will explore how they can be of clinical value either to help in diagnosis, prognosis, or even treatment of endometriosis (Table 2).

**Table 2:** Immune cell characteristics and clinical relevance in endometriosis CD = cluster of differentiation, IL = interleukin, TNF-α = tumor necrosis factor-alpha, TGF-β = transforming growth factor-beta, VEGF = vascular endothelial growth factor, IFN-γ = interferon gamma, CXCL10 = C-X-C motif chemokine ligand 10, NETs = neutrophil extracellular traps, RORγt = retinoic acid receptor-related orphan receptor gamma t, FoxP3 = forkhead box P3, PD-1 = programmed cell death protein 1, PD-L1 = programmed death-ligand 1, CSF-1R = colony-stimulating factor 1 receptor, MPO = myeloperoxidase, DCs = dendritic cells, imDC = immature dendritic cells, mDC = mature dendritic cells, PDC = plasmacytoid dendritic cells, MDC1 = myeloid dendritic cell type 1, MDC2 = myeloid dendritic cell type 2, Th17 = T helper 17 cells, Treg = regulatory T cells, MCs = mast cells, TRPV1 = transient receptor potential vanilloid 1, NK = natural killer

Immune Cell Type	Key Markers / Subtypes	Major Cytokines	Functional Role	Clinical Relevance
Macrophages	CD68, CD206 (M2), CD163	IL-6, TNF-α, VEGF, TGF-β1	Phagocytosis, angiogenesis, fibrosis	Lesion growth, pain; potential target: CSF-1R inhibitors (Lv et al., 2024)
NK Cells	CD56^dim/CD16^hi, CD56^bright	IFN-γ (↓), IL-10, TGF-β	Immune surveillance, cytotoxicity	Reduced cytotoxicity; checkpoint inhibition with PD-1 blockade (Walankiewicz et al., 2023)
Neutrophils	CD66b, MPO	IL-8, VEGF, CXCL10	Early inflammation, NETs, angiogenesis	Associated with pain severity and inflammatory microenvironment
DCs	CD1a (imDC), CD83 (mDC), CD303 (PDC), CD141 (MDC2)	IL-10, IL-6, IL-1β	Antigen presentation, immune tolerance	Skewed toward tolerogenic phenotype; under study for DC modulation therapies
T Helper 17 (Th17)	RORγt, IL-17A	IL-17, IL-22	Promote inflammation, inhibit Treg	Associated with lesion persistence, IL-17 inhibition as therapy (Yu et al., 2023)
Treg	FoxP3, CD25^high, CD4^+	IL-10, TGF-β	Immune suppression, tolerance	Downregulated in lesions; key in immune escape
B Cells	CD19, CD20	IL-6, IFN-γ	Autoantibody production	Contribute to inflammation and autoimmunity; less central role
Mast Cells	Tryptase, Chymase	TNF-α, Histamine, IL-6	Promote neurogenesis, degranulation	Cluster around nerves; may be targeted with mast cell stabilizers

Role of the innate immune system in endometriosis

The innate immune system is the body's first line of defense against bacteria or any foreign material. Thus, it makes sense that in the case of endometriosis, it would also be the first responder to the ectopic endometrial tissue [16,17]. That is why understanding how each of the cellular components of the innate response functions is important in the pathogenesis of endometriosis.

Macrophages

The most abundant immune cells in the endometrial lining are the macrophages, which play a vital role in the menstrual cycle. For instance, they participate in repairing and regenerating the endometrial lining during the proliferative and secretory phases and also contribute to the shedding of the endometrial wall during menses [35]. For instance, during the proliferative phase, the macrophages express a cluster of differentiation 54 agonists (CD54), CD69, and CD71, which are activation and adhesion molecules, to stimulate the repair and regeneration of the endometrial lining [35]. During the secretory phase, they secrete VEGF, which regulates gland remodeling and angiogenesis [35]. Finally, during menses, when they are most abundant, they secrete matrix metalloproteinase (MMP), MMP-9, MMP-12, and MMP-14, which cause the shedding of the endometrial lining [35]. Macrophages also phagocytose and degrade menstrual debris and refluxed endometrial tissue during menstruation to sustain homeostasis [17].

In endometriosis, the number of macrophages is significantly increased in the peritoneal cavities of patients [17]. Macrophages are recruited to the peritoneal cavity of patients with endometriosis in response to the presence of ectopic endometrial tissue, tissue damage, and various chemokines, such as IL-17A, monocyte chemotactic protein-1 (MCP-1), IL-8, and chemokine (C-C motif) ligand 5 (CCL5), to clear menstrual debris and refluxed heme-iron lesions [35-38]. Macrophages that are attracted to the site of endometriosis have varying origins. Some come from the refluxed endometrial tissue; others are macrophages already present in the peritoneal cavity, and others are derived from the bone marrow. Since they come from different locations, each has different functions [36]. For example, macrophages coming from the refluxed endometrial tissue have been linked to enhancing the formation of endometriosis; as a result, they are regarded as pro-endometriosis [36]. On the other hand, macrophages derived from the bone marrow reduce the number of endometriosis lesions and increase the number of embryo-derived large peritoneal macrophages (LpM) of the endometrial macrophages, which also reduces the number of endometriosis lesions. Hence, these types of macrophages protect against the formation of endometriosis [36].

After being recruited to the site of an endometriosis lesion, the macrophages can be classified into M1 if activated by the classic pathway, or M2, if activated by the alternative pathway. M2 macrophages are thought to help in the development, growth, and vascularization of the ectopic endometrial lesion (pro-endometriosis), while M1 macrophages do not [17,37]. One paper suggested that ectopic endometrial stromal cells secrete fractalkine (FKN), which increases the level of IL-10 but decreases the level of IL-2, causing M1 macrophages to shift to M2 to enhance the formation of endometriosis [39]. Another paper supported the idea of M1 to M2 polarization but by the Smad2/Smad3 pathway [40]. However, the exact balance between the subsets of macrophages is not clearly understood, as some papers suggest that the level of M1 macrophages is higher in endometriosis compared to M2, while others argue that there shouldn't be two separate categories of the types of macrophages since they can interact and generate complex and mixed phenotypes [17,37,41].

**Figure 3:**
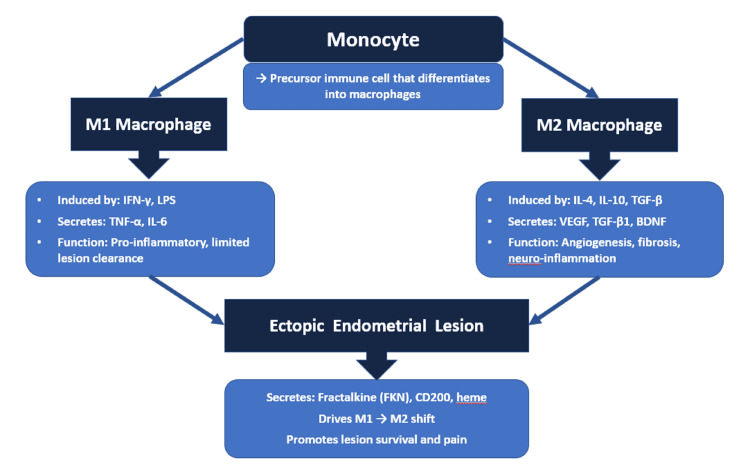
Macrophage Polarization in Endometriosis IFN-γ = interferon-gamma, LPS = lipopolysaccharide, IL-4 = interleukin-4, IL-10 = interleukin-10, TGF-β = transforming growth factor-beta, TNF-α = tumor necrosis factor-alpha, IL-6 = interleukin-6, VEGF = vascular endothelial growth factor, TGF-β1 = transforming growth factor-beta 1, BDNF = brain-derived neurotrophic factor, FKN = fractalkine, CD200 = an immunomodulatory molecule, heme = an iron-containing compound Image Credit: Authors

Macrophages contribute to the pathogenesis of endometriosis in four ways: reduction in their phagocytic ability, promotion of chronic inflammation through oxidative stress, enhancement of angiogenesis of the endometrial lesions, and induction of fibrogenesis (Figure 3).

Reduction in the Phagocytic Ability of the Macrophages

Under normal circumstances, when endometrial tissue is refluxed into the peritoneal cavity, the macrophages clear any menstrual debris. Unfortunately, in endometriosis, the macrophages cannot phagocytose this menstrual debris [42]. This happens due to the following:

Increased level of heme: Macrophages attempt to consume the ectopic endometrial lesions, which contain old red blood cells (RBCs), also known as heme. Heme contains iron, which can either be stored in the form of ferritin or hemosiderin in macrophages or bind to the iron transporter, transferrin (Tf) [43,44]. Macrophages consume high levels of heme, or iron, which impairs their phagocytic ability, thus enhancing the development of endometriosis [45].

Signaling pathway: Another way in which the phagocytotic ability of macrophages is decreased is through the upregulation of the signal regulatory protein-α (SIRP-α). Essentially, the endometrial-like tissue releases SIRP-α, which directly decreases the phagocytic ability of the macrophage and attenuates the expression of CD36. CD36, a scavenger receptor, aids macrophages in removing oxidized LDL and dead neutrophils [17,37,46].

Prostaglandin E2: It also plays a role in decreasing the phagocytic ability of macrophages by further repressing the expression of CD36 and decreasing the level of matrix MMP-9 [17]. In addition to that, the ectopic endometrial tissue expresses CD200, which binds to the CD200 receptor on the macrophages, essentially telling it, "Don't eat me," thus enhancing the formation of endometriosis by preventing phagocytosis [3].

Promotion of chronic inflammation​​​​​​: As mentioned earlier, macrophages consume the old RBCs, which contain heme. Heme impairs the phagocytic ability of the macrophages. Moreover, their iron content causes iron-mediated damage and oxidative stress. This leads to tissue injury and damage and contributes to the chronic inflammatory state, which helps in the survival of the ectopic endometrial tissue and causes pain. Macrophages produce reactive oxygen species, which lead to the formation of nuclear factor-kappa B (NF-kB), which is a crucial component in the pathophysiology of endometriosis and in pain induction [35,37].

Enhancement of angiogenesis: Macrophages can create new blood vessels in areas where there is low oxygen being delivered to the tissue, as in endometriosis. CD 206+ macrophages, classified as M2 macrophages, are known to encourage the formation of new blood vessels around the endometriosis lesion [47]. They do so by secreting VEGFA and fibroblast growth factor 2 (FGF-2), which promote the formation of new blood vessels, as well as matrix metalloproteinases MMP-2 and MMP-9, which degrade the existing extracellular matrix to create space for new blood vessels that nourish the endometriotic lesions [35,37]. A recent study suppressed the level of macrophages through a drug called clodronate in mice affected with endometriosis, resulting in a decrease in the size of the ectopic endometrial tissue growth and angiogenesis suppression [48].

Fibrosis formation: In addition to enhancing the processes of chronic inflammation and angiogenesis, macrophages also promote the process of fibrogenesis. One way in which this could happen is through a specific type of macrophage called M2a. When IL-4 activates M2a macrophages, it causes them to release tissue growth factor beta-1 (TGF-β1). TGF-β1 stimulates the process of fibrogenesis through the SMAD3 pathway [49-51]. Another way fibrosis is induced is through a molecule found in the peritoneal fluid called sphingosine 1-phosphate (S1P), which causes the switching of macrophages to M2, resulting in the release of IL-6 and cyclooxygenase-2 (COX-2), thereby enhancing inflammation and scar formation. IL-6 and soluble IL-6 receptor (sIL-6R) defective signaling lead to constant activation of signal transducer and activator of transcription 3 (STAT3) and NF-kB, which stimulate further scar formation by producing excessive collagen in the eutopic endometrial stromal cells [52]. In addition to that, macrophages produce numerous pro-inflammatory and pro-fibrotic mediators like TNF-α, IL-1, IL-6, IL-8, VEGF, and lysyl oxidases (LOXs), which stimulate and facilitate fibrosis [53].

Macrophages not only help endometriosis to grow and develop they also play a role in inducing pain related to endometriosis through several mechanisms, such as driving neuroinflammation, cross-talking with the nerve fibers, production of neurotrophic factors, causing peripheral and central sensitization, and causing oxidative stress [16,18,30]. Nerves in the endometriosis lesion release chemicals like colony-stimulating factor-1 (CSF-1), chemokine ligand 2 (CCL-2), leukemia inhibitory factor (LIF), and pancreatitis-associated protein III (PAP-III), which induce the migration of macrophages toward the nerve ending [18,30,35,36]. In addition to that, they cause macrophages to produce neurotrophic factors such as brain-derived neurotrophic factor (BDNF), neurotrophin-3 (NT-3), and insulin-like growth factor-1 (IGF-1), which cause neurogenesis in the endometriosis lesions, sensitization of the nerve ending, and upregulation of the perception of pain [30]. IG-1 is one of the key mediators of pain associated with endometriosis, and its levels correspond to the level of pain. It does this by increasing the expression of nociceptive genes and enhancing sensitization through the upregulation of the transient receptor potential cation channel (TRPV1) and other nociceptive ion channels, causing nerve cells to become more easily excited, which leads to a lower pain threshold and hyperalgesia [30]. Macrophages also release inflammatory cytokines, IL-1β, IL-6, and TNF-α, prostaglandins, and chemokines, Fractalkin (CX3CL1), causing peripheral sensitization by cross-talking with the sensory neuron, heightening the pain sensitivity, and causing chronic inflammation [16,29,46]. As the process of chronic inflammation and peripheral sensitization continues, the brain becomes trained to respond to these signals in a heightened way, leading to central sensitization [54,55].

Macrophages play a clinically essential role in the pathophysiology of endometriosis and endometriosis-related pain. For example, removing macrophages using liposomal clodronate in mouse models decreased the size of the endometriosis lesion and reduced the pain [30]. Also, using linsitinib, an IGF-1 inhibitor, has been shown to reduce nerve growth and activation of nociceptive genes, thus relieving the pain [30]. However, future research is required to understand the different subsets of macrophages and how they contribute to the pathogenesis of endometriosis and to explore the feasibility of using macrophage markers as diagnostic and targeted therapies for women with endometriosis.

Mast cells

Mast cells (MCs) are key players in innate immunity, playing an essential role in inflammatory and immune processes. They release preformed and newly synthesized mediators, including histamine, chemotactic factors, and various proteases such as peroxidase, tryptase, and chymase, as well as tumor necrosis factor-alpha (TNF-α), which influence the tissue environment [56]. Additionally, MCs synthesize and secrete a large number of newly formed mediators, including prostaglandins, leukotrienes, and numerous cytokines (e.g., interleukins IL-1, -3, -4, -5, -6, -10, -14, and 17), along with trophic factors such as nerve growth factor (NGF) [57]. These mediators modulate the inflammatory response and the activation of other immune cells, including neutrophils, eosinophils, macrophages, and lymphocytes [58].

MCs play a dual role: on one hand, they contribute to immune system activation and defense against pathogens; on the other, they are involved in regulating and resolving inflammation to prevent tissue damage and promote the restoration of homeostasis [59]. In fertile women, maximum degranulation of MCs and a massive release of pro-inflammatory mediators occur during each menstrual cycle. MCs contain hormone receptors and are responsive to estrogen since they express estrogen receptors, but they do not express progesterone receptors. They play a physiological role in regenerating the endometrium [60]. For instance, in the proliferative phase, estrogen stimulates the enrollment and degranulation of MCs, thus increasing their number in the uterine cavity. However, they do not actively play a significant role during the proliferative phase. They are essentially on standby during the proliferative phase [61]. In the secretory phase, the progesterone levels begin to rise, and the MCs become activated. Although lacking the progesterone receptor, it is suggested that the cells in the endometrium indirectly cause activation of the MCs [61]. Once activated, they release tryptase, chymase, and TNF-α. TNF-α causes the activation of macrophages, which enhances their role in this phase. During the menstrual phase, MCs play a vital role by releasing inflammatory cytokines, like TNF-α, and proteases, like MMP, which contribute to the shedding of the endometrium [61].

Despite the important role of MCs in the normal menstrual cycle, the number of MCs significantly increases around the blood vessels and nerve cells of the endometriotic lesions [60]. In endometriosis, the level of estrogen and substance P increases, which leads to the recruitment and activation of MCs. MCs release pro-inflammatory cytokines like TNF-α and IL-6, which contribute to the inflammatory environment in endometriosis, leading to the progression of the disease and pain [61-63]. In addition, TNF-α and IL-6 cause further recruitment and activation of the MCs, leading to a vicious cycle [61].

In addition to secreting these pro-inflammatory cytokines, MCs tend to cluster around nerve endings in endometriotic lesions and secrete additional pro-inflammatory cytokines like histamine, tryptase, PGE2, TNF-α, and NGF, leading to hyperalgesia. MCs also promote angiogenesis and neurogenesis around the endometriotic lesion, further supporting its growth and causing dysmenorrhea and dyspareunia [63,64].

Some studies used MC stabilizers like ketotifen, which demonstrated a decrease in the size of the endometriosis lesion and the pain associated with it in mice [65]. Although not tested yet, another study also suggested combining GnRH analogs, and MC-targeted treatment could produce a synergistic effect in decreasing the pain associated with endometriosis [62]. Further studies are encouraged to test the applicability of these findings.

Natural killer cells

NK cells, a type of lymphocyte that is part of the innate immune system, are mainly found in the blood, liver, spleen, lungs, liver, and gut [66]. In the endometrium, a specialized subset of NK cells called uterine natural killer (uNK) cells plays a role in the normal menstrual cycle and early pregnancy [66,67]. uNK cells differ from the NK cells in the peripheral blood in their phenotypic and functional properties because they express different chemokine receptors and adhesion molecules, resulting in a different migratory response. In addition, the number of uNK cells is influenced by the level of progesterone in the body. For instance, in the proliferative phase of the menstrual cycle, where progesterone levels are low, there are few numbers of agranular uNK cells. After ovulation, the progesterone level rises, leading to a significant increase in the number of uNK cells. In the mid or late secretory phase, uNK cells increase further in number and become granular. They also interact with the spiral uterine arteries, preparing the uterus for implantation if fertilization occurs. If fertilization does not happen, the level of progesterone drops, as well as the number of uNK cells, due to a decrease in soluble factor products, which maintain vascular integrity, and the endometrial lining sheds, resulting in menstruation [66,67]. However, if fertilization occurs, the level of progesterone rises due to the maintenance of the corpus luteum, resulting in decidual natural killer cells (d-NK), which are the most common immune cells present in early pregnancy. d-NK cells play a role in early pregnancy by preparing the endometrium for placentation through the production of angiogenic factors that cause mucosal vascularization, regulating trophoblastic invasion in the uterine wall by being close to the extra-villous trophoblast, secreting growth factors that promote fetal development, and playing a role in immune tolerance between the mother's immune system and fetal antigens [68,69].

During menses, any refluxed endometrial tissue in the peritoneum is typically cleared by the NK cells. However, this does not happen in endometriosis because NK cells are dysfunctional [70-72]. For instance, the cytotoxic activity of the NK cells is impaired because there is upregulation in the killer inhibitory receptors (KIR) and downregulation of the killer activating receptors (KAR) [17]. An example of a KIR is CD94/NK group 2 member A (NKG2A) on NK cells, along with the leukocyte immunoglobulin-like receptor subfamily B member 1 (LILRB1). When the endometrial cells express the human leukocyte antigen G (HLA-G), it binds to the LILRB1 receptor, which sends inhibitory signals to the NK cells [72]. An example of KAR receptors includes NKG2D, which is suppressed by platelet-derived TGF-β1, a cytokine, thus impairing the cytotoxic ability of the NK cells [17]. Platelets and other inhibitory cytokines, IL-6, IL-16, IL-17, IL-1β, IL-10, and TGF-β, are found in the peritoneal fluid in patients with endometriosis and function to prevent the NK cells from degranulation and producing interferon (IFN)-γ, impairing the NK cell function. The macrophages produce IL-10 and TGF-β by interacting with the ectopic endometrial stromal cells, thereby inhibiting the degranulation of the NK cells and the secretion of IFN-γ and perforins [73]. Moreover, ectopic endometrial tissue expresses programmed death-ligand 1 (PD-L1), which is upregulated by estrogen. When the programmed death-1 (PD-1), receptor on the NK cells, binds to PD-L1, it diminishes the NK cell function, escaping the immune system and allowing the endometriosis lesions to grow and survive [74]. Endometriosis lesions not only express PD-L1 but also express major histocompatibility complex (MHC) class I on their cells, which, when bound to the killer-cell immunoglobulin-like receptors (KIR) on the NK cells, activate the inhibitory system of the NK cells. This allows the lesions to evade immune surveillance and survive [17]. Estrogen also plays a direct role in impairing the function of the NK cells. Primarily, they prevent the endometrial stromal cells from autophagy by inhibiting the CXCL12/CXCR4 pathway, making it harder for the NK cells to eliminate them [75]. Moreover, it impairs the function of the NK cells by activating STAT3, which reduces the levels of an important molecule for the function of the NK cell called hematopoietic cellular kinase (HCK). As a result, the levels of IL-8 and IL-23, proinflammatory cytokines, promote the arrest of the NK cell's function [76]. Finally, estrogen stimulates the endometrial stromal cells to secrete IL-5, which decreases the level of granzyme B, IFN-y, and NKG2D, also making the NK cells unable to eliminate the ectopic endometrial tissue [77]. 

The pathophysiology of how NK cells play a role in endometriosis-associated pain is not clear. However, specific subtypes of NK: CD56HiCD16dim NK cells, which are found in the peripheral blood, and CD8+CD56dimCD16Hi NK cells, which are found in the peritoneal fluid, are found to be elevated. For instance, the level of CD8+CD56dimCD16Hi NK cells in the peritoneal fluid was increased in patients with endometriosis compared to normal patients, and the level of the increase correlated with the severity of the dysmenorrhea. Patients complaining of rectorrhagia showed increased levels of CD56+ CD4 T cells and CD56+ CD4 T cells while having lower levels of CD8+ NK cells and a lower ratio of CD56dimCD16Hi NK cells to CD56HiCD16dim NK cells. Patients complaining of dyschezia showed lower circulating NK cell percentages, particularly CD8+ CD16+ CD56+ NK cells in the PF of severe dyschezia cases [78].

More research is required to assess the feasibility of using the level of CD56HiCD16dim NK cells as a non-invasive biomarker for diagnosing and testing the severity of endometriosis [72,79]. Further research is encouraged to explore the potential of targeting specific molecules such as LILRB1 and HLA-G to enhance the cytotoxic activity of NK cells. Additionally, the use of IL-2 or BCG to boost NK cell function warrants investigation [72,79].

Neutrophils

Neutrophils, a type of granulocyte, are normally present in very low amounts throughout the entire menstrual cycle. However, they typically increase in number in the inflammatory stage just before menses begin due to chemokine release by MCs, macrophages, and other cells [80]. They account for 6-15% of the cells present during the inflammatory state. They contribute to the process of endometrial shedding by releasing matrix MMPs and activating other matrix MMPs, which leads to the breakdown of the extracellular matrix and hence menses [80]. They also aid in repairing the endometrial lining after menses, as shown by a study conducted on mice where the absence of neutrophils hindered the process of endometrial tissue repair [81]. In addition, neutrophils also help in the implantation and maintenance of pregnancy by secreting IFN-γ, VEGF, and IL-17, which promote cell division and angiogenesis [80].

In endometriosis, the number of neutrophils is significantly increased in the blood, peritoneal fluid, ectopic, and ectopic endometrium. The high number of neutrophils in the peritoneal fluid and ectopic endometrial tissue is mainly due to the high levels of chemotactic agents such as growth-regulated gene alpha, interleukin 8, epithelial neutrophil-activating peptide-78 (ENA-78), and human neutrophil peptides-1, 2, and 3 [81]. Once neutrophils reach the peritoneal fluid and ectopic endometriosis lesions, they participate in the inflammatory process of endometriosis by secreting pro-inflammatory cytokines like IL-8, VEGF, and C-X-C motif chemokine ligand 10 (CXCL-10), all of which enhance the progression of the disease [80-82]. IL-8 is not only a chemotactic attractant for neutrophils, but it also contributes to the adhesion of the endometrial cells and helps create a lesion [80]. Neutrophils also secrete IL-17A, which induces the cells of the endometrial-like tissue to produce CXCL-1, which attracts more neutrophils and causes them to produce the pro-inflammatory cytokines mentioned earlier, contributing to the microenvironment that attracts even more neutrophils and supports the ectopic endometrium [81,83]. Moreover, neutrophils are a source of VEGF, which contributes to the high level of VEGF in the peritoneal fluid, creating an inflammatory microenvironment and promoting angiogenesis. This allows the ectopic endometrial-like tissue to develop further and promotes adhesion of other endometrial tissue, causing more endometriosis. Recent research also shows that neutrophil extracellular traps (NETs) are elevated in the peritoneal fluid of patients with endometriosis; however, their contribution to the pathogenesis of endometriosis is still not clear [16,17,81].

Neutrophils not only play a role in the pathogenesis of endometriosis, but they also play an important role in the pain associated with endometriosis by releasing multiple cytokines and activating pain-related pathways [82,84]. IL-8 not only helps attract more neutrophils to the site of endometriosis, but it also helps initiate the inflammatory cascade, which is part of the process of pain induction related to endometriosis. Additionally, higher levels of ENA-78 in the peritoneal fluid of patients with endometriosis are associated with more severe pain. Neutrophils also indirectly cause neuroinflammation by secreting factors that recruit macrophages to the end of nerve fibers in endometriosis lesions [84,85]. As a result, macrophages secrete CSF-1 and estradiol (E2) that lead to the production of BDNF and NT-3, which promote nerve growth in the ectopic endometrial tissue and cause sensitization, leading to increased pain [30]. Peripheral sensitization also occurs when the neutrophils release inflammatory cytokines like IL-8, TNF-alpha, and prostaglandin E2, which activate the peripheral nerve endings. Neutrophils can also stimulate the macrophages to release IL-1b, a proinflammatory cytokine, which stimulates the release of neurotrophic factors, such as NGF and BDNF, that, in turn, induce new nerve fibers that are directly linked to endometriosis lesions, overstimulating the sensory neurons and causing pain [84-86]. Due to the consistent stimulation of the peripheral nerve fibers by the neutrophils and the cytokines released by it because of the constant inflammatory state, the brain begins to sense pain even though there is no stimulation happening in the peripheral nerve ending, through a process known as central sensitization [80].

A lot of research has been done to try to find ways to correlate the level of neutrophils in blood with the severity of the disease [80,82,87]. Many papers found that combining the neutrophil-to-lymphocyte ratio (NLR) with CA-125 can help in diagnosing endometriosis in a non-invasive way [80,81,87]. However, some papers did not report any relation between NLR and endometriosis, prompting further research to determine whether this correlation can be used as a diagnostic marker for endometriosis [80,88]. Some sources also suggest that preventing the production of the inflammatory cytokines produced by neutrophils can be a pharmacological target for the treatment of endometriosis-related pain.

Dendritic cells

DCs are a specialized type of antigen-presenting cell that take up self and foreign antigens and present them to other cells of the immune system, both innate and adaptive [89,90]. Once the DCs engulf, process, and present the antigen, they can produce cytokines that activate an immune response and other cytokines that promote immune tolerance, depending on the type of antigen presented. In the context of the menstrual cycle, DCs play a role in menstruation, tissue repair, angiogenesis, and embryo implantation by secreting relevant chemokines and cytokines [91]. There are different types of DCs, and each plays a role in the menstrual cycle. First off, we have immature dendritic cells (imDC), which express the immune marker CD1a and are found in the peripheral blood. They are responsible for engulfing and processing the antigens and are the most abundant DCs in the uterus. They have strong migration ability, but they cannot present the antigens to T cells and activate them; hence, they play a role in immune tolerance [90,91]. On the other hand, mature dendritic cells (mDC), whose immune marker is CD83, are responsible for antigen presentation and are fewer in number compared to the imDC in the uterus. Unlike imDC, they can activate the T cells, leading to an immune response. Later-stage mDC, represented by the immune marker DC-LAMP+, is a special subset of mDC that also has a role in the menstrual cycle [90,91]. In addition, plasmacytoid dendritic cells (PDCs]), which have CD303 as their immune marker, are adept at the formation of type 1 IFN and inducing tolerance [92]. Finally, myeloid dendritic cells (MCDs) present the antigens to T cells using MHC II. MDCs have 2 types: MDC1, which express CD1c, and MDC2, which express CD141 [90,92]. The number of DCs increases in the uterus as the menstrual cycle progresses. In the proliferative phase, imDC concentration is the same in the basal and functional layer of the endometrium, assisting in immune surveillance and tissue homeostasis. At the same time, the level of MC1 increases in the blood. Between the proliferative and secretory phases, PDC cell levels increase [90,92]. During the secretory phase, imDC increases more in the basal layer than in the functional layer as the endometrium prepares itself for potential implantation. In addition, DCs produce cytokines that help in tissue remodeling and angiogenesis. Between the secretory phase and the menstrual phase, DC-LAMP+ increases significantly to participate in tissue breakdown and antigen presentation [90,92]. In the menstrual phase, the level of imDC and DC-LAMP+ increases dramatically in the basal layer. DCs in this phase also produce MMPs to stimulate the breakdown and shedding of the endometrial tissue, resulting in menstruation [90,92].

Unlike the other cells of the innate immune system, the exact role of DCs in the pathogenesis of endometriosis is still not fully clear [17]. Some studies show that the level of DCs in endometriosis does not change compared to healthy patients, but what differs is the subtype of DC. In endometriosis, the level of imDC increases while the mDC cells decrease [93,94]. Also, some studies show that removing the DCs in mouse models of endometriosis leads to an increase in lesions [95,96], while other studies show it leads to a decrease, but this depends on the stage of the disease [97]. However, what is known so far is that the level of imDC is more pronounced in endometriosis lesions. They contribute to the pathogenesis of endometriosis by secreting IL-10, a tolerogenic cytokine, which promotes the migration of ectopic endometrial tissue and promotes angiogenesis, protecting the ectopic endometrial tissue from immune clearance [17,91]. As mentioned earlier, they are not able to activate the T-cells, which promotes a state of immune tolerance that supports the growth of the endometriosis lesion [91]. Compared to imDCs, the level of the mDCs is markedly decreased, hence why they fail to present the antigens to the T cells properly, resulting in weaker activation of the T-cells and a stunted immune response [91]. To highlight their role, scientists injected lipopolysaccharide in mouse models with endometriosis, which causes the number of mDCs to increase, leading to a decrease in size in the endometriosis [91]. Finally, although the level of MCD 1, which expresses high levels of mannose receptor, is more abundant in endometriosis, they can phagocytose dead endometrial stromal cells, but they produce pro-inflammatory cytokines IL-1β and IL-6, which not only promote inflammation but also help the endometriosis lesion to grow and thrive [16,98].

Although DCs do not directly contribute to the pain associated with endometriosis, they may do so indirectly by secreting cytokines that support lesion survival, such as IL-10, and pro-inflammatory cytokines like IL-1β and IL-6, which promote neurogenesis. This effect is mediated through an increased presence of iDCs and their interaction with macrophages, ultimately contributing to pain [17,99]. Moreover, the combination of the pro-inflammatory environment and the production of nerve fibers both increase the perception of pain, possibly due to the downregulation of the opioid receptors [99].

Although there is a lot to know about the role of DCs in the pathogenesis of endometriosis, it is clear that they play a role in its pathogenesis by altering the number of their subtypes, producing inflammatory cytokines, and promoting angiogenesis and neurogenesis. Therefore, using the information we have about DC, we can conduct studies to help us in diagnosing and treating endometriosis. For instance, measuring the number of imDC or even the cytokines produced by the DC, like IL-1β, IL-6, and IL-10, in the peritoneal fluid of patients with endometriosis might serve as a diagnostic tool. Discovering ways to inhibit or decrease the number of imDC or even to increase the number of mDC may prevent endometriosis from developing, inhibit angiogenesis and neurogenesis, decrease the size of the endometriosis lesion, and prevent pain [91,99].

Role of the adaptive immune system in endometriosis

After the innate immune system fails to eliminate the endometriosis lesion, antigen-presenting cells activate the adaptive immune system, trying to eliminate the ectopic tissue. Thus, the cell-mediated and humoral components both play a role in the pathogenesis of endometriosis [100].

T lymphocytes (specifically regulatory T cells, T helper 17 cells, cytotoxic T cells)

T cells play an important role throughout the menstrual cycle as well as in the early stages of pregnancy. Their number as well as their subtypes changes, both in the blood and the endometrial cavity, throughout the cycle based on the changes in hormone levels [101-103]. For instance, in the proliferative phase, CD8+ T cells, which are cytotoxic, increase in number to help remove the remaining endometrial tissue and pathogen from the previous menstrual cycle. CD450+, a type of memory T cell, is also increased in this phase to regulate the immune response and prepare the endometrium for possible implantation [102]. Then, the level of Th1 cells rises in the proliferative phase to participate in tissue remodeling and endometrial regeneration. CD3+ and CD4+ T cells reach their highest quantity in the blood during the early proliferative phase, playing a role in the immune balance systemically [103]. Then, in the secretory phase, there is an observed elevation of the number of CD30+ T cells in the endometrial cavity, which are required to regulate the immune response to create a tolerogenic immune environment to permit implantation of the embryo if fertilization happens. On the contrary, the level of CD8+ T cells decreases in number due to the decrease in progesterone, to prevent embryo rejection by reducing their cytotoxic activity. Th2 and Treg cells both rise in number to enhance immune tolerance to allow prospective embryo implantation [102,103]. The level of Th17 cells, which are pro-inflammatory cells, remains stable. Otherwise, an excess of them will impair the implantation of the embryo. At the same time, if they increase in number, they might cause endometrial inflammation; as a result, they are crucial in the role of endometriosis [101]. Mid-secretory phase, the progesterone level reaches its peak, leading to a further decrease in the immune response. Consequently, the levels of CD3+ and CD4+ T cells in the blood decrease [103]. During the menstrual phase, the progesterone level drops dramatically, and the lining of the endometrium begins to shed. Hence, the levels of CD8+ T cells and Th1 cells increase again to assist in the shedding of the endometrium and prevent any infections. On the contrary, the levels of Treg cells drop to allow the immune system to clear the menstrual debris [103].

Dysregulation in the number and subtypes of T cells, especially the Treg, Th17, and CD8+ T cells, all play a vital role in the pathogenesis of endometriosis. However, most of the studies done to date use different techniques, such as flow cytometry and immunohistochemistry, and take samples from different sites, such as peritoneal fluid and endometrial lesion, which causes a large amount of discrepancy between the papers. As a result, the findings may contradict each other [104]. As mentioned earlier, the Treg cells play a role in immune suppression. In endometriosis, Treg cells, although functional, promote the development and growth of the ectopic endometrial tissue by preventing the immune system from being activated [104]. CCL20 enhances the migration of Treg cells to the endometriosis lesion [105]. When they migrate to the site of the ectopic endometrial lesion, they secrete IL-10 and TGF-β, which downregulate the immune response, allowing the endometriosis lesion to escape [31]. Activated Treg (FOXP3⁺⁺CD45RA⁻), a subtype of Tregs distinct from resting Tregs (FOXP3⁺CD45RA⁺), prevents the maturation of imDCs by secreting IL-10. This suppresses the immune response, which may contribute to immune tolerance in conditions like endometriosis. Nonetheless, one paper eliminated the Treg cells from mouse models with endometriosis, which led to inflammation and angiogenesis and supported the growth and development of the endometrial lesion [17]. Although the exact role of Th17 cells is not understood in the pathogenesis of endometriosis, a lot of studies state that the macrophages and the ectopic endometrial cells secrete IL-27, which attracts the Th17 to the site of these lesions and causes them to secrete IL-10, which promotes the growth of the endometriosis [104]. Th17 also secretes IL-17, which promotes angiogenesis and pro-inflammatory cytokines, further enhancing the progression of endometriosis and pain [12]. Also, the level of Th17 cells is increased in endometriosis patients, and the level of increase correlates with the severity and stage of the disease [33]. The role of CD8+ T cells is not clearly understood either, but the studies suggest that they play a role in the pathogenesis of endometriosis. Many studies show that there is no difference in the number of CD8+ T cells in the blood. However, the levels of CD8+ T cells in the peritoneal fluid of endometriosis patients are higher in some studies and lower in other studies [17,106]. In addition, some studies even demonstrate that the cytotoxic ability of the T lymphocytes is reduced because the endometriosis lesions express FAS ligand, which binds to the FAS receptor on the CD8+ T cells and causes them to undergo apoptosis [106,107]. However, more research is required to help us understand the role of T cells in the pathogenesis of endometriosis by addressing these underlying discrepancies in the data.

The exact mechanism of how T cells play a role in endometriosis-related pain is not quite clear, but it can be implied. For example, since the Treg cells allow the endometriosis lesions to survive and grow, they facilitate disease progression and can cause more pain. However, the direct mechanism is yet to be deduced [78,104]. Th17 cells secrete a pro-inflammatory cytokine, IL-17, and prevent the maturation of the DCs, both of which can cause pain related to endometriosis [12].

Since more research needs to be done to better understand the role of T cells in endometriosis and the pain associated with it, there is no clear-cut evidence about how T cells can be used in the clinical setting. However, based on the studies we have can try to analyze the levels of the T cell subtypes in the peritoneal fluid, although it is invasive. More research should be done to find a non-invasive diagnostic method. Also, by finding a way to modulate the immune environment and regulate the levels of the T cell subtypes, we can find a way to treat endometriosis effectively [100,104,106,108].

B lymphocytes

The level of B cells in the endometrium, which are part of the adaptive immune system that secrete immunoglobulins, is influenced by hormonal fluctuations throughout the menstrual cycle. Thus, they play a role throughout the menstrual cycle [109]. Generally, the levels of the B cells in the peripheral blood tend to remain stable throughout the cycle, but they fluctuate in the endometrium [102,109,110]. In the proliferative phase, when the estrogen level is high, the B cells are present, but not abundant, and they help in immune surveillance and tissue repair [102]. In the secretory phase, the level of the B cells, specifically CD20+, begins to rise and increase in activity to promote immune tolerance to prepare the uterus for a potential pregnancy [109,110]. The role of the B cells in the menstrual phase is not quite understood, but it is speculated that there is a temporary rise in their level to possibly help in the shedding and breakdown of the endometrium, help in clearing menstrual debris, and prevent infection [102,109].

Although the exact role of B cells in the pathogenesis of endometriosis is still not clear and is still under investigation, research shows that the number of B cells is increased both in the blood and the peritoneal fluid of patients with endometriosis [111]. B cells, stimulated by estrogen, also secrete autoantibodies like anti-endometrium, anti-DNA, antiphospholipid, and antinuclear antibodies, which, although their mechanism is not clear, play a role in the pain and infertility associated with endometriosis [112]. It has also been demonstrated that the level of anti-endometrial antibodies correlates with the severity of the disease [24]. In addition, they also produce some pro-inflammatory cytokines like IL-6, IL-17, and IFN-γ, which also play a role in the pathogenesis of endometriosis by creating an inflammatory environment and recruiting other cells, including macrophages, to enhance the growth of the lesion [17]. Research also shows that despite the increase in the number of B cells, their activity has decreased through the promotion of the PD-1/PD-L1 pathway, which normally inhibits the immune response, thus allowing the endometriosis lesion to survive [113].

Moreover, the exact mechanism by which B cells contribute to the pain associated with endometriosis is not clearly understood. However, the presence of the pro-inflammatory cytokines and the autoantibodies contributes to the inflammatory environment in endometriosis, which might lead to pain [17,112]. Also, the presence of the inflammatory environment, in general, leads to the production of NGF, which causes neurogenesis in the endometriosis lesion, leading to pain [17]. More research is encouraged for us to understand the role of B cells in the pain associated with endometriosis.

Since the level of anti-endometrial antibodies is related to the severity of the disease, they could be used as a non-invasive diagnostic marker [17]. Moreover, one research paper experimented on mice where they administered Bruton's tyrosine kinase inhibitors, such as ibrutinib, and the size of the endometriosis lesion decreased. Ibrutinib also decreases inflammation by decreasing the level of IL-6 and mRNA expression of COX-2. In addition, it helped decrease the activation of B cells in the spleen and peritoneal cavity; on the other hand, it increased the expression of Breg cells, which are a type of B cells that decrease the inflammatory response and promote tolerance. In the same study, they also gave anti-CD20 and CD20, which led to the depletion of all subsets of B cells, including the Breg cells. As a result, there was no decrease in the endometriosis lesion or even the inflammation. Researchers suggest that because the Breg cell was depleted by the anti-CD20 treatment, it could be the reason why the anti-CD20 treatment did not have any effect on endometriosis [111].

Future directions

Emerging insights regarding the immune mechanisms involved in endometriosis present encouraging possibilities for application in clinical settings. Future research should focus on validating immune-related biomarkers (e.g., IL-17, PD-1, CSF-1R) for early diagnosis and personalized treatment approaches. Ongoing trials targeting inflammatory and immune checkpoint pathways highlight the potential of immunomodulation as a therapeutic strategy. The incorporation of single-cell technologies and spatial transcriptomics will be key to enhancing personalized treatment strategies and identifying new drug targets.

## Conclusions

The immune system plays a vital role in the normal menstrual cycle. Disruption in the immune system plays a role in the establishment, development, growth, and progression of endometriosis. Furthermore, immune dysregulation causes a state of chronic inflammation, which also plays a role in the dysmenorrhea associated with endometriosis. Potential biomarkers have been hypothesized to be used in the clinical setting to help in the diagnosis and even predict the prognosis of endometriosis. Some immune targets have also been suggested to be used in the treatment of endometriosis. However, further research is needed before they can be applied to the clinical setting.
